# Purification Effect of Water Eutrophication Using the Mosaic System of Submerged–Emerged Plants and Growth Response

**DOI:** 10.3390/plants13040560

**Published:** 2024-02-19

**Authors:** Baoliang Chang, Yingchun Xu, Ze Zhang, Xiaowen Wang, Qijiang Jin, Yanjie Wang

**Affiliations:** 1 Key Laboratory of Landscape Agriculture, Ministry of Agriculture and Rural Affairs, Key Laboratory of Flower Biology and Germplasm Development, Ministry of Agriculture and Rural Affairs, Key Laboratory of Biology of Ornamental Plants in East China, National Forestry and Grassland Administration, College of Horticulture, Nanjing Agricultural University, Nanjing 210095, China; changbl9601@163.com (B.C.); xyc@njau.edu.cn (Y.X.);; 2 CAS Key Laboratory of Forest Ecology and Management, Institute of Applied Ecology, Chinese Academy of Sciences, Shenyang 110016, China; 3 Liaoning Shenyang Urban Ecosystem Research Station, National Forestry and Grassland Administration, Shenyang 110164, China

**Keywords:** plant combination, urban water bodies, eutrophication, bioremediation, submerged plant, water purification

## Abstract

Aquatic plants play a crucial role in the sustainable management of eutrophic water bodies, serving as a valuable tool for water purification. However, the effectiveness of using aquatic plants for improving water quality is influenced by landscape considerations. In practical applications, challenges arise concerning low purification efficiency and compromised aesthetic appeal when utilizing plants for water purification. To address these issues, this study aimed to examine the impact of aquatic plants on the purification of simulated landscape water bodies, specifically focusing on the effectiveness of the mosaic system of submerged–emerged plants in remediating eutrophic water bodies. Our findings indicated that individual aquatic plants exhibited limited efficacy in pollutant (total nitrogen, total phosphorus, ammonia nitrogen, and chemical oxygen demand) removal. However, when combined in appropriate proportions, submerged plants could enhance species growth and improve the purification efficiency of polluted water bodies. Notably, the mosaic system of submerged–emerged plants neither significantly promoted nor inhibited the growth of each other, but it effectively removed pollutants from the simulated water bodies and inhibited turbidity increase. The comprehensive evaluation ranked the purification capacity as *Canna indica*-submerged plants combination (C + S) > *Thalia dealbata*-submerged plants combination (T + S) > *Iris pseudacorus*-submerged plants combination (I + S) > *Lythrum salicaria*-submerged plants combination (L + S). Both C + S and T + S configurations effectively mitigated the rise of water turbidity and offered appealing landscape benefits, making them viable options for practical applications in urban landscape water bodies. Our study highlights that a submerged–emerged mosaic combination is a means of water purification that combines landscape aesthetics and purification efficiency.

## 1. Introduction

Urban landscape water bodies are facing a serious issue of eutrophication due to the rapid development of cities and the frequent human activities occurring within them [1,2,3]. Eutrophication is a natural process of organic matter and nutrient enrichment through succession in a water body, which may be accelerated and exacerbated by human nutrient inputs, resulting in the deterioration of water quality. The main pollutants in water during this process are nitrogen, phosphorus, and organic matter. The main test indicators are total nitrogen (TN), total phosphorus (TP), ammonia nitrogen (NH_3_-N), and chemical oxygen demand (COD) [4,5]. The acceleration of urbanization has intensified the eutrophication of water bodies, which not only damages the quality of water, but also affects the overall landscape aesthetic of water bodies (reduced transparency, algae growth, and unpleasant odors). In severe cases, it can even pose risks to human health (drinking inadvertently), as urban landscape water bodies often serve as a common point of contact with water sources [6,7].

Phytoremediation has gained consensus as a low-cost and efficient restoration technology, representing a crucial aspect of sustainable water body development [8,9,10]. The application of plants for water treatment has been extensively studied and implemented in urban landscape water management [11,12]. The use of emerged plants (*Triarrhena lutarioriparia*, *Miscanthus sinensis*, *Zizania caduciflora*, *Thalia dealbata*, *Vetiveria zizanioides*, and *Acorus calamus*) can effectively improve water quality and reduce nitrogen and phosphorus concentrations in water bodies. The use of submerged plants (*Myriophyllum spicatum*, *Hydrilla verticillata*, *Vallisneria* sp., and *Ceratophyllum demensum*) also has a significant improvement effect on water quality [13,14]. However, some studies have found that plant combinations have a stronger purification effect compared to individual plant species [15,16]. Plant combinations tend to provide higher purification rates and higher stability based on potential synergistic effects [17].

Aquatic plants can be broadly categorized into four life types: submerged plants, free-floating plants, floating-leaved plants, and emerged plants [18,19]. It should be noted that not all combinations of aquatic plants from different life types display synergistic effects, and certain combinations fail to significantly improve water-purification efficiency [20]. Therefore, the identification of efficient phytoremediation assemblage models that effectively address eutrophication in water bodies is of utmost importance. The mosaic system of submerged–emerged plants (hereinafter referred to as the mosaic system) has been studied and proven to exhibit favorable synergistic effects in the purification of polluted water bodies [21,22].

To ensure the sustainable development of urban landscape water bodies, it is imperative to explore efficient and scientifically grounded plant combination patterns for managing and maintaining the overall environment of these water bodies [4,23]. An ideal management approach for urban landscape water bodies involves enhancing the purification effects of submerged plants while considering the landscape aesthetics of emerged plants. We assume that different biomass ratio combinations of submerged plant combinations may be a way to improve purification efficiency.

The findings from this research will advance our understanding of the role of aquatic plant assemblages in pollutant purification and provide valuable insights for managing eutrophic water bodies in urban areas using mosaic systems. The objective was to examine the purification effects of mosaic systems. Therefore, our experiment was divided into two phases. The first phase of this study involved selecting two submerged plants (*C. demersum* and *M. verticillatum*) and four emerged plants (*Iris pseudacorus*, *Canna indica*, *Lythrum salicaria*, and *T. dealbata*) to compare their individual purification capacities. Subsequently, we compared the purification effect and growth of submerged plant combinations with different biomass ratios in eutrophic polluted waters. In the second phase, mosaic systems were created by combining the most effective submerged plant combinations for purification with four species of emerged plants known to have good landscaping effects.

## 2. Results

### 2.1. The Purification of Water Quality by Single Species of Plants

**Figure 1:**
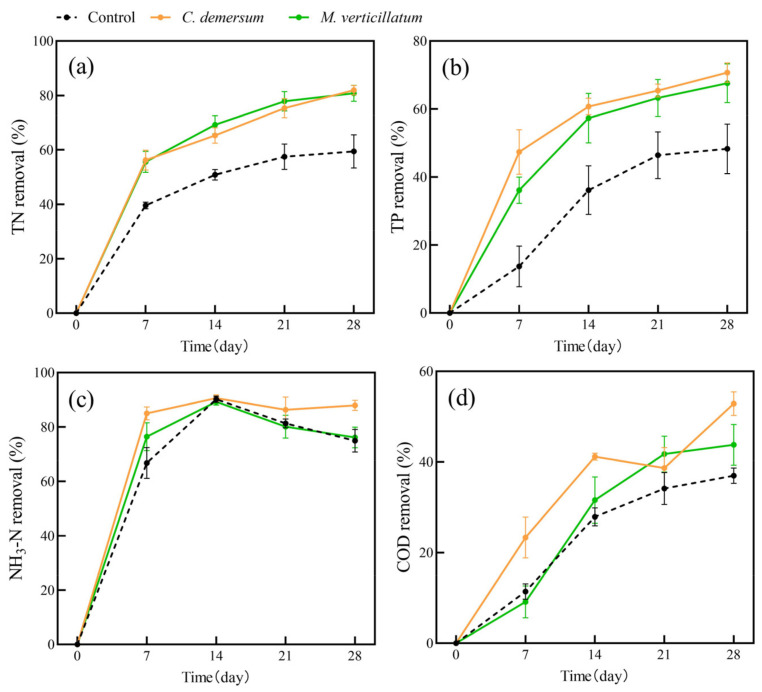
The removal rates of TN (**a**), TP (**b**), NH_3_-N (**c**), and COD (**d**) from polluted water bodies by submerged plants. Values are means ±SD (*n* = 3).

**Figure 2:**
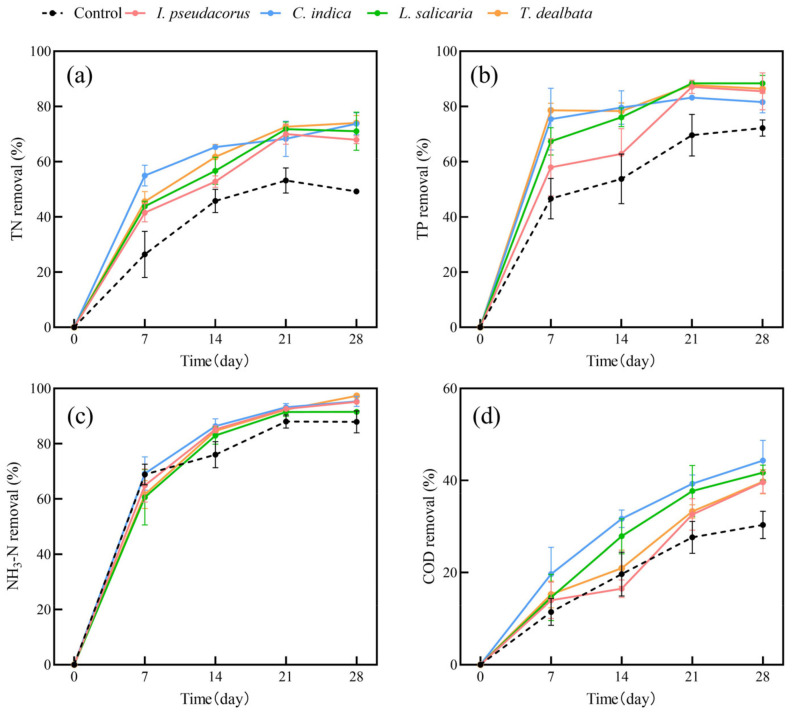
The removal rates of TN (**a**), TP (**b**), NH_3_-N (**c**), and COD (**d**) from polluted water bodies by emerged plants. Values are means ±SD (*n* = 3).

In this study, we conducted an investigation into water purification using single plant species (Figure 1 and Figure 2). It is evident that both the two submerged plants and four aquatic plants exhibited improved removal of water pollutants compared to the plant-free control group. The removal rates of water pollutants by the plant groups increased gradually over time. In submerged plants, *C. demersum* has a higher removal rate of TP than *M. verticillatum* in all stages. In terms of NH_3_-N and COD removal, *C. demersum* demonstrated relatively favorable performance, with removal rates of 88% and 53%, respectively. *M. verticillatum* was the next highest, with removal rates of 76% and 44%, respectively. The removal effects of the four emerged plants on TN, TP, and COD were not significantly different from each other, but they were all superior to the control group.

Our results showed that the average removal rates of TN, TP, NH_3_-N, and COD by submerged plants were 82%, 69%, 82%, and 48%, respectively. For emerged plants, the average removal rates of TN, TP, NH_3_-N, and COD were 71%, 86%, 95%, and 41%, respectively. Both submerged and emerged plants showed significantly higher removal rates for TN and TP than the control group. However, the removal of NH_3_-N and COD by submerged plants showed fluctuation. The trends in pollutant-removal rates were similar for the four emerged plants.

### 2.2. Growth Interaction and Purification Effect of Submerged Plant Combinations with Different Proportions

#### 2.2.1. Relative Yield Totals (RYT)

**Figure 3:**
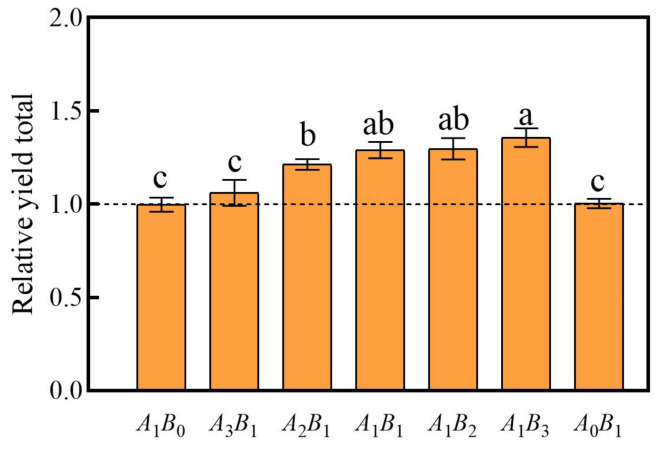
Relative yield totals for the treatment group. A: *C. demersum* B: *M. verticillatum*. Numbers indicate biomass proportions. The value above the dashed line indicates that there is a promoting effect among species. Different lowercase letters indicate significant differences at *p* < 0.05 level.

The RYT values can be used to clarify whether there is a mutual promotion or mutual inhibition of growth between the two plants. In our study, all five combinations of *C. demersum* and *M. verticillatum* demonstrated an overall promotion of growth (Figure 3). All combinations, except for A_3_B_1_, significantly promoted growth, with the highest promotion observed in the A_1_B_3_ combination.

#### 2.2.2. The Purification Effect of Different Plant Combinations

**Figure 4:**
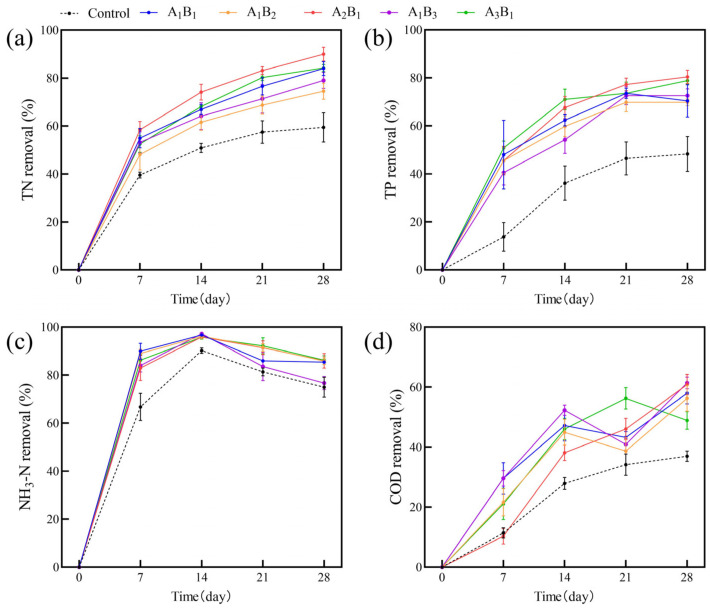
The removal rates of TN (**a**), TP (**b**), NH_3_-N (**c**), and COD (**d**) from polluted water bodies in each treatment group. Values are means ±SD (*n* = 3). A: *C.demersum* B: *M. verticillatum*. Numbers indicate biomass proportions.

The impacts of varying ratios of submerged plants on the removal of TN, TP, NH_3_-N, and COD from the water column are depicted in Figure 4. Each combination of plants exhibited a progressive increase in pollutant-removal rates compared to the control group without plants. The removal rates of TN, TP, and NH_3_-N rapidly increased in the early part of the experiment and then stabilized, consistently surpassing the control group, and the removal rate of COD showed fluctuation. Among them, the removal rate of NH_3_-N decreased in the later stage of the experiment. Over the course of the 28-day experiment, A_2_B_1_ exhibited the most effective TN and TP removal, while multiple plant ratios demonstrated optimal removal efficiency for NH_3_-N and COD.

Mutual-growth promotion resulting from different biomass ratios enhances overall biomass, subsequently influencing the purification efficiency of plants. We utilized the entropy method to evaluate the purification ability of different ratios of submerged plant combinations with weights of 23%, 34%, 5%, and 38% for TN, TP, NH_3_-N, and COD, respectively. The results showed that the A_2_B_1_ ratio had the highest purification effect with a combined score of 75.38 (Table 1).

### 2.3. Growth Interaction and Purification Effect of Mosaic Systems

**Figure 5:**
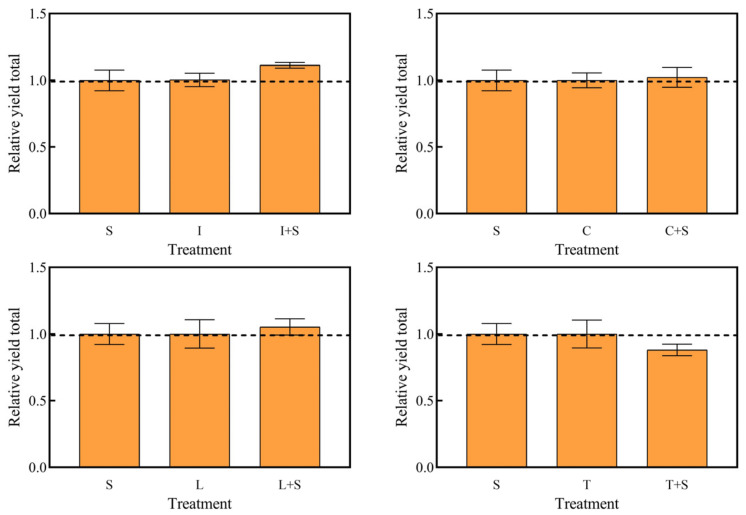
Relative yield totals for each treatment group. The value above the dashed line indicates that there is a promoting effect among species. Values are means ±SD (*n* = 3). I: *I. pseudacorus*, C: *C. indica*, L: *L. salicaria*, T: *T. dealbata*, S: submerged plants group.

The interactions between the different mosaic systems of emerged plants and submerged plants are shown in Figure 5. The RYT of the four mosaic systems was not significant compared to the submerged plant group (S) and the monoculture of emerged plants group, indicating that the mosaic system as a whole did not show significant promoting or inhibitory effects.

**Figure 6:**
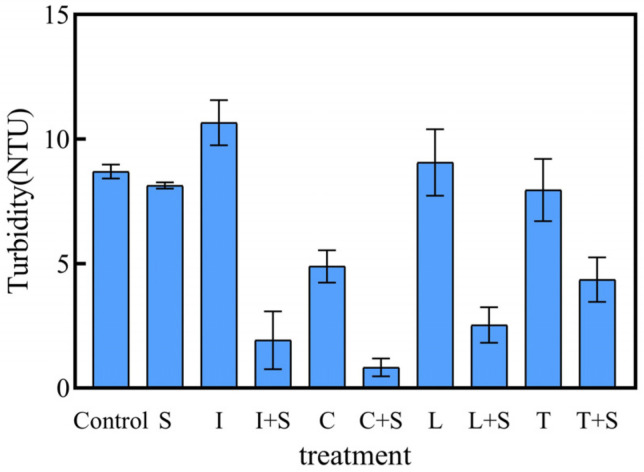
Turbidity of polluted water in different treatments on the 28th day. Values are means ±SD (*n* = 3). NTU, nephelometric turbidity unit. I: *I. pseudacorus*, C: *C. indica*, L: *L. salicaria*, T: *T. dealbata*, S: submerged plants group.

Turbidity is a direct reflection of water clarity in lakes and can inhibit the recovery of plant communities by affecting plant photosynthesis [24]. Furthermore, appropriate aquatic plant establishment can effectively mitigate turbidity in water bodies [25]. Surprisingly, our 28-day experimental results revealed that mosaic systems had a significant purification effect on turbidity (Figure 6). Water turbidity was significantly lower in all mosaic systems compared to their respective monoculture-emerged plant treatments and the control groups (*p* < 0.05). Among them, the C + S treatment group demonstrated the lowest turbidity level of 0.82 NTU in the water column by day 28.

**Figure 7:**
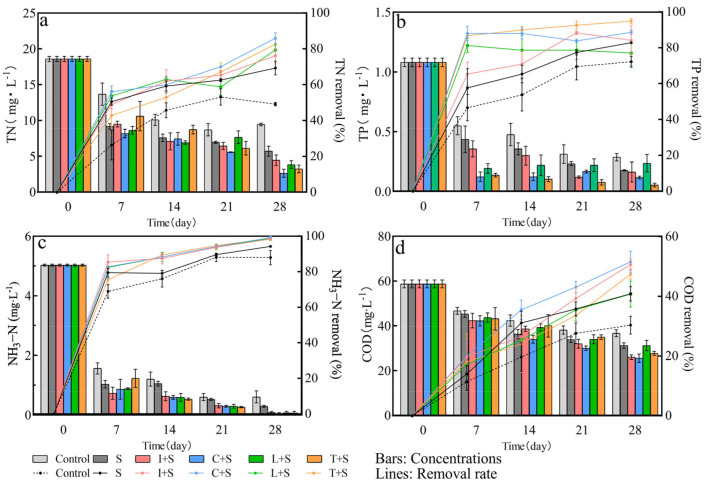
Changes in pollutant concentration and removal rate in polluted water bodies under different plant configuration patterns (**a**) TN; (**b**) TP; (**c**) NH_3_−N; (**d**) COD. Values are means ±SD (*n* = 3). I: *I. pseudacorus*, C: *C. indica*, L: *L. salicaria*, T: *T. dealbata*, S: submerged plants group.

The impact of different plant mosaic patterns on the removal of TN, TP, NH_3_-N, and COD in the water column is presented in Figure 7. Additionally, the mixed cultivation of aquatic plants enhances pollutant-removal efficiency through species interactions [26]. Among the four mosaic systems evaluated, the C + S configuration demonstrated effective purification of TN with a removal rate of 86%. Furthermore, the T + S configuration exhibited notable purification of TP with an 89% removal rate. However, no optimal combination was identified for NH_3_-N and COD purification. The removal of NH_3_-N by all four mosaic systems reached more than 98%. In the case of COD, the removal rate for C + S and I + S was the highest, at around 50%.

### 2.4. Comprehensive Evaluation of Purification Capacity

The comprehensive evaluation of mosaic systems using the entropy method revealed that all four mosaic systems outperformed the submerged plant combinations, resulting in an improved purification efficiency (Table 2). The weights of TN, TP, NH_3_-N, and COD are 43%, 11%, 3%, and 43%, respectively. Among them, the C + S configuration attained the highest overall evaluation score, followed by T + S. Throughout the trial, the plants exhibited normal growth and flowering, indicating their potential for water restoration and landscape applications.

## 3. Discussion

### 3.1. Differences in Purification by Submerged Plants

As a result of the study, it was found that different species within the same life type demonstrated varying purification capacities, consistent with previous findings [27]. Submerged plants undergo their main life cycle underwater and rely heavily on the water environment for growth and development. Submerged plants, being well-adapted to eutrophic environments and fully immersed in water, generally exhibit greater pollutant-purification capacities compared to emerged plants [28]. We found that there was high TN and TP removal by both submerged plants. This could be attributed to the leaf morphology of submerged plants. Aquatic plants such as *C. demersum* and *M. verticillatum* possess large specific surface areas, which facilitate effective eutrophication control [29]. It is worth noting that even the plant-free control group achieved a high removal rate of NH_3_-N, which may be attributed to variations in inorganic nitrogen concentration caused by nitrification and denitrification in the water column [30]. Similarly, in a 75-day experiment conducted by Cui et al. [31] with *M. verticillatum*, the inorganic nitrogen concentration in the plant-free control water rapidly decreased to nearly zero values. This trend may be attributed to the presence of free ammonia and ammonium ions, which readily dissociate from the water matrix [32]. Moreover, aquatic plants exhibit a certain preference for ammonia nitrogen absorption [33,34].

This study revealed differences in the removal efficiency of water column pollutants with different combinations of plant proportions, which may be attributed to changes in total biomass in the system, and more biomass leads to higher purification efficiency [35,36]. However, in the A_1_B_1_, A_1_B_2_, and A_1_B_3_ ratios, the species interaction further intensified, without a commensurate increase in the purification effect. This indicates that excessive biomass does not augment the purification effect any further, consistent with the findings of Hu et al. [37]. Their research on the optimal biomass of aquatic plants for lake ecological restoration highlighted that maintaining a biomass of 5.5 kg·m^−2^ is crucial, as an excessively high biomass fails to continue promoting the restoration process.

### 3.2. Differences in Purification by Mosaic Systems

Nutrients present in highly eutrophic water bodies can induce synergistic or competitive interactions among different plant species [38]. However, compared with the submerged plants group and single emerged plants, none of the four mosaic systems in this experiment exhibited significant overall promotion or inhibition (Figure 5). This lack of significant effects may be attributed to environmental factors that influence plant growth, as demonstrated by Li et al. [39]. In their study, the interspecific relationship between *C. demersum* and *Phragmites australis* was primarily influenced by changes in the relative growth rate of *C. demersum* and the population density of *P. australis* along a nitrogen gradient. The substantial reduction in pollutant content within the water body can be attributed to the well-developed root systems of water-holding plants, which directly absorb pollutants such as nitrogen and phosphorus, while also facilitating the flocculation and sedimentation processes [40].

The gradual increase in the pollutant-removal rate observed in each mosaic configuration over time, surpassing that of the submerged plant group, can be attributed to the comprehensive utilization of distinct ecological spaces within the mosaic. These findings align with the studies by Thongtha et al. [41] and Su et al. [42]. However, the purification effect of different mosaic systems on water bodies exhibited some variations, likely due to the response of microorganisms in the water body to different plant combinations [43]. Microbial nitrification and denitrification reactions represent the main denitrification pathways within the three microcosms studied [44,45]. Additionally, the abundance of aquatic plant species enhances the diversity of microorganisms within the water body, thereby promoting nitrogen and phosphorus degradation by microorganisms [46]. Eventually, plant harvesting will facilitate the removal of nutrients from the water body [47]. Notably, the L + S configuration obtained a relatively low comprehensive score during the experiment, possibly attributed to the presence of apoplast, which could lead to secondary water body pollution through its decomposition [48,49].

The mosaic systems have limited inhibitory effects on water turbidity. The significant turbidity inhibition observed in C + S treatment may be attributed to the extensive root system of *C. indica*, facilitating the adsorption of suspended substances and consequently improving water quality [50]. In conclusion, the combination of multiple plant species remarkably impedes the escalation of turbidity in eutrophic water bodies, maintaining a clearer water state more effectively than individual plant species alone. These findings align with the study conducted by Zhang et al. [51].

## 4. Materials and Methods

### 4.1. Materials

Submerged plants: We selected *Ceratophyllum demensum* (“A”) and *Myriophyllum verticillatum* (“B”), typical pollution-resistant submerged plants in Nanjing, China. Emerged plants: The emerged plants with a high frequency of landscape application in Nanjing, China, such as *Iris pseudacorus* (I), *Canna indica* (C), *Lythrum salicaria* (L), and *Thalia dealbata* (T), were purchased from Mu Yang Wanshui Flower Nursery. The roots were washed with deionized water before both experiments. Vigorously growing plants of a similar size were selected for the experiments.

Experimental water: The water quality of urban landscape water bodies in Nanjing was investigated in the early stages, and we set the corresponding concentrations for the main indicators of eutrophication. Set three conventional indicators for nitrogen and phosphorus pollutants were as follows: TN, TP, and NH_3_-N. In addition, COD represents the oxygen content required for the complete oxidation of all organic matter in the water body. To reflect the organic matter situation in the water body, we choose COD as the indicator for the organic matter content. Water was obtained from the river near the experimental site. Then, NH_4_Cl, NaH_2_PO_4_, KNO_3_, and C_6_H_12_O_6_ were used to regulate the levels of each pollutant in the water bodies of all experimental groups to the set levels. The concentrations of TN, TP, NH_3_-N, and COD in the water were 18.58 mg·L^−1^, 1.08 mg·L^−1^, 5.03 mg·L^−1^, and 58.70 mg·L^−1^ at the beginning of the experiment.

### 4.2. Methods

The experiments were conducted in August 2020 and August 2021 at the Whitehorse site of Nanjing Agricultural University. Plants with good and consistent growth were selected as the test material after the plants were incubated in water for 7 days. We used 46 cm standard diameter polyethylene non-porous culture pots as planting containers and spread washed quartz sand on the bottom of the containers to fix submerged plants. The whole experiment was set-up in a rain shelter to exclude the interference of natural rainfall.

The first phase of the experiment was set-up to compare the purification capacity of a single plant species. The total biomass of the submerged plants was 180 g; we set-up separate planting groups for *C. demersum* and *M. verticillatum*. In addition, five submerged plant configuration ratios were set, with *C. demersum* and *M. verticillatum* biomass ratios of 1:3 (A_1_B_3_), 1:2 (A_1_B_2_), 1:1 (A_1_B_1_) 2:1 (A_2_B_1_), and 3:1 (A_3_B_1_), respectively. The second phase of the trial set up four mosaic systems, the water-holding plants were two mature plants of similar seeding plant height and health: *I. pseudacorus*-submerged plants group (I + S), *C. indica*-submerged plants group (C + S), *L. salicaria*-submerged plants group (L + S), and *T. dealbata*-submerged plants group (T + S), respectively, and set *I. pseudacorus* group (I), *C. indica* group (C), *L. salicaria* (L), *T. dealbata* (T), and submerged plants group (S). The submerged plant group was the optimal biomass ratio combination of the two submerged plants in the first phase of the experiment. The total biomass of the submerged plant assemblage was 90 g and that of the water-holding plants was 2 plants. The eutrophic water bodies without plants were set as the control group in both experiments. Each treatment was replicated three times for 28 days of the experiment.

### 4.3. Sampling and Testing Methods

We used syringes to take 50 mL of water samples mixed with 100 mL of water samples from each of the upper and lower layers of the water body at 7 d intervals in both tests, which were then filtered through a microporous filter membrane (0.45 μm) and used for water-quality testing. After each sampling, we replenished the barrel with deionized water to maintain the water level in the barrel to compensate for the reduced water due to natural factors and water sample collection during the test. The fresh weight of the plants was measured at the end of the experiment on the 28th day after sucking out the water from the exterior of the plants. The collected water samples were transferred to the laboratory for chemical analysis. The COD values of the samples were measured using fast-elimination spectrophotometry; the NH_3_-N content of the samples was determined using the spectrophotometric method using Na reagent; the TP was analyzed using the ammonium molybdate spectrophotometer method; and the TN concentration was determined using the alkaline potassium persulfate digestion-UV spectrophotometer method.

In addition, for urban water bodies, turbidity can reflect the clarity of the water body, which directly affects people’s senses. We found that in the first phase of the experiment, plants had a great influence on the turbidity of the water body. So, in the second phase of the experiment, we diverted our attention to the turbidity of the test water body. Turbidity was measured using a portable turbidimeter. The unit of turbidity is Nephelometric Turbidity Units (NTU). Because eutrophication causes an increase in turbidity, the second phase of the experiment focused only on the inhibitory effect of plants on turbidity. The initial concentration of turbidity was not specifically set. The concentration was 1.3 NTU.

Complex interactions occur between plants that grow normally in the same environment. It has been shown that the relative yield total (RYT) can indicate whether species interactions promote or inhibit turbidity [52]; Equation (1) was used to calculate the RYT.

(1) $$RYT=\frac{1}{2}\times \left(\frac{Y_{i}}{P_{i}Y_{ai}}+\frac{Y_{j}}{P_{j}Y_{aj}}\right)$$

where *Y_i_* is the biomass of plant *i* in the plant assemblage; *Y_j_* is the biomass of plant *j* in the plant assemblage; *P_i_* is the percentage of plant *i* in the plant combination; *P_j_* is the percentage of plant *j* in the plant combination; *P_i_* + *P_j_* = 1. *Y_ai_* is the biomass of plant *i* when planted alone; and *Y_aj_* is the biomass of plant *j* when planted alone. When RYT = 1, the interactions between species were unclear; when RYT > 1, the species showed mutual promotion; and when RYT < 1, the species showed mutual inhibition. Monocultures in the plant combinations were used as a control group with RYT = 1.

Equation (2) was used to calculate the removal rate of each water-quality indicator:

(2) $$P=\frac{M_{0}-M_{i}}{M_{0}}\times 100\%$$

where M_0_ is the initial concentration and M*_i_* is the indicator concentration of the water column on day 28.

We used the entropy value method [53] to determine the weights of water-quality indicators, and finally, we conducted a comprehensive evaluation of the purification effect of the submerged–emerged plant mosaic combination.

### 4.4. Statistic Analysis

Experimental data analysis was performed using Excel 2019 and SPSS 19.0 software. One-way ANOVA was used for statistical and correlation analyses of data, Pearson test was performed at 0.05 probability level (*p* < 0.05), and data were expressed as mean ± standard deviation. GraphPad Prism 8 software was used for graphing.

## 5. Conclusions

In conclusion, this study demonstrates the effectiveness of aquatic plants in removing pollutants from the water column, with the removal rate exhibiting a gradual increase over time compared to the plant-free control group. Furthermore, an optimal biomass ratio in the combination of submerged plants was found to promote mutual growth and enhance the purification effect, while an inappropriate biomass ratio hindered purification efficiency. The comprehensive evaluation results indicated that *C. demersum* and *M. verticillatum* had the best purification effect with an initial biomass ratio of 2:1. The experiments involving mosaic systems demonstrated that well-designed mosaic systems significantly improve the purification capacity of water bodies. Different plant combinations exhibited favorable purification effects on eutrophic water bodies, allowing for the selection of suitable plant species based on specific water body characteristics to maximize purification advantages. The comprehensive evaluation ranked the purification capacity as C + S > T + S > I + S > L + S. Both T + S and C + S configurations effectively mitigated the rise of water turbidity, and offered appealing landscape benefits, making them viable options for practical application in urban landscape water bodies.

## Figures and Tables

**Table 1:** Comprehensive evaluation of water-purification capacity of submerged plant combination.

Treatments	A Score of Each Indicator				Total Score	Rank
	TN	TP	NH_3_-N	COD		
A_2_B_1_	20.58	27.50	4.11	23.19	75.38	1
A_1_B_3_	18.07	24.84	3.66	23.41	69.98	2
A_1_B_1_	19.20	24.09	4.08	22.11	69.48	3
A_3_B_1_	19.26	26.97	4.12	18.64	68.99	4
A_1_B_2_	17.05	23.88	4.10	21.46	66.49	5
Control	13.59	16.52	3.58	14.10	47.80	6

Note: A: *C. demersum*, B: *M. verticillatum*. Numbers indicate biomass proportions.

**Table 2:** Comprehensive evaluation of the purification capacity of mosaic systems.

Treatments	A Score of Each Indicator				Total Score	Rank
	TN	TP	NH_3_-N	COD		
C + S	36.94	9.75	2.97	22.17	71.84	1
T + S	35.51	10.43	2.96	20.37	69.27	2
I + S	32.72	9.26	2.95	21.79	66.72	3
L + S	34.12	8.47	2.96	17.54	63.09	4
S	29.80	9.11	2.83	17.57	59.31	5
Control	21.15	7.94	2.64	13.05	44.79	6

Note: I: *I. pseudacorus*, C: *C. indica*, L: *L. salicaria*, T: *T. dealbata*, S: submerged plants group.

## Data Availability

Data recorded in the current study are available in all tables and figures of the manuscript.
